# Alterations in Perivascular Sympathetic and Nitrergic Innervation Function Induced by Late Pregnancy in Rat Mesenteric Arteries

**DOI:** 10.1371/journal.pone.0126017

**Published:** 2015-05-07

**Authors:** Esther Sastre, Javier Blanco-Rivero, Laura Caracuel, María Callejo, Gloria Balfagón

**Affiliations:** 1 Departamento de Fisiología, Facultad de Medicina, Universidad Autónoma de Madrid, Madrid, Spain; 2 Instituto de Investigación Sanitaria IdiPAZ, Madrid, Spain; State University of Rio de Janeiro, Biomedical Center, Institute of Biology, BRAZIL

## Abstract

**Background and Purpose:**

We investigated whether pregnancy was associated with changed function in components of perivascular mesenteric innervation and the mechanism/s involved.

**Experimental Approach:**

We used superior mesenteric arteries from female Sprague-Dawley rats divided into two groups: control rats (in oestrous phase) and pregnant rats (20 days of pregnancy). Modifications in the vasoconstrictor response to electrical field stimulation (EFS) were analysed in the presence/absence of phentolamine (alpha-adrenoceptor antagonist) or L-NAME (nitric oxide synthase-NOS- non-specific inhibitor). Vasomotor responses to noradrenaline (NA), and to NO donor DEA-NO were studied, NA and NO release measured and neuronal NOS (nNOS) expression/activation analysed.

**Key Results:**

EFS induced a lower frequency-dependent contraction in pregnant than in control rats. Phentolamine decreased EFS-induced vasoconstriction in segments from both experimental groups, but to a greater extent in control rats. EFS-induced vasoconstriction was increased by L-NAME in arteries from both experimental groups. This increase was greater in segments from pregnant rats. Pregnancy decreased NA release while increasing NO release. nNOS expression was not modified but nNOS activation was increased by pregnancy. Pregnancy decreased NA-induced vasoconstriction response and did not modify DEA-NO-induced vasodilation response.

**Conclusions and Implications:**

Neural control of mesenteric vasomotor tone was altered by pregnancy. Diminished sympathetic and enhanced nitrergic components both contributed to the decreased vasoconstriction response to EFS during pregnancy. All these changes indicate the selective participation of sympathetic and nitrergic innervations in vascular adaptations produced during pregnancy.

## Introduction

Pregnancy is associated with a decrease in systemic vascular resistance that, despite the marked increase in blood volume and cardiac output, maintains or reduces maternal blood pressure, in both experimental animals and humans. Adaptations to pregnancy have been studied in several vascular beds, but the mechanisms underlying the altered vessel function are complex and only partially understood.

Vascular adaptations to pregnancy include both an endothelium-dependent pathway associated with increased production of vasodilators [1] and an endothelium-independent pathway associated with altered vasomotor smooth muscle cell responses to different vasoactive substances [2,3,4], that decrease myogenic reactivity [5] and increase vascular compliance [6]. However, activation of additional endothelium-independent pathways has been strongly suggested [7]. Perivascular innervation has a significant influence on peripheral vascular resistance involving the sympathetic, cholinergic, nitrergic, peptidergic and/or sensory innervations, which are specific to the vascular bed under consideration.

The mesenteric artery plays a pivotal role in global peripheral resistance in rats, especially in pregnancy; during this physiological process, mesenteric perfusion is strongly increased. These arteries are innervated by sympathetic nerves, which mediate vasoconstriction mainly via noradrenaline (NA) release, but also by nitrergic innervation, which induces vasodilatation by nitric oxide (NO) release, and sensory innervation through release of the vasodilator calcitonin gene-related peptide neuropeptide (CGRP) [8,9,10]. Electric field stimulation (EFS) produces a vasomotor response that is the integrated result of the effect of these different neurotransmitters [10]. The alterations in the functional roles of these components have been associated with changes in synthesis, release, response and/or metabolism of the different neurotransmitters in several physiological and pathological circumstances [11,12,13,14].

Neuronal adaptation to pregnancy by mesenteric arteries it has been reported to be time-dependent. In late pregnancy diminished sympathetic nerve-mediated constriction has been associated with a decreased vasoconstrictor response to NA [7], while possible changes in NA release have been suggested but not investigated [4]. No changes have been reported in sensory innervation [4] but there is an increased vasodilation to CGRP [7,15,16]. It is widely known that estrogens modulate vascular tone activating endothelial nitric oxide synthase (eNOS) and several studies have reported that vascular adaptation in pregnancy is associated with an increase in eNOS protein expression [17,18,19]. In previous studies we have observed that changes in levels of sex steroids are associated with changes in nitrergic innervation function [14,20]. However, to the best of our knowledge, the possible role of nitrergic innervation in vascular adaptations to pregnancy remains unexplored.

Taking these data into account, we considered it relevant to study possible simultaneous changes in the different kinds of perivascular innervation during pregnancy, consequently the aim of this work is to analyze whether the possible functional changes in sympathetic, nitrergic and sensory innervations in late pregnancy could be associated with the decreased vascular resistance observed in the mesenteric artery, as well as the mechanisms that may be implicated.

## Materials and Methods

### Animals

Female Sprague-Dawley rats (4–6 months old) were obtained from the Animal Quarters and housed in the Animal Facility of the Universidad Autónoma de Madrid (registration number EX-021U) in accordance with guidelines 609/86 of the E.E.C., R.D. 233/88 of the Ministerio de Agricultura, Pesca y Alimentación of Spain, and the *Guide for the Care and Use of Laboratory Animals* published by the United States National Institute of Health [NIH publication No. 85–23, revised 1996]. All experimental procedures involving animal use were approved by the Ethics Committee of the Universidad Autónoma de Madrid. Rats were housed at a constant room temperature, humidity, and light cycle (12:12 h light-dark) with free access to tap water and fed with standard rat chow *ad libitum*, and were divided into two groups: Control (virgin females in oestrous phase) and pregnant rats (20 days of pregnancy). The stage of the oestrous cycle was determined by examination of vaginal smears; oestrous females exhibited the presence of cornified cells. The day when a vaginal plug was found was considered to be day 1 of pregnancy.

Animals were sacrificed by CO_2_ inhalation followed by decapitation; superior mesenteric artery was carefully dissected, cleaned of connective tissue and placed in Krebs-Henseleit solution (KHS, in mmol/L: NaCl 115, CaCl_2_ 2.5, KCl 4.6, KH_2_PO_4_ 1.2, MgSO_4_.7H_2_O 1.2, NaHCO_3_ 25; glucose 11.1; Na_2_EDTA 0.03) at 4°C. For protein expression analysis, some segments were rapidly frozen in liquid nitrogen and kept at -80°C until the day of analysis.

### Vascular reactivity

The method used for isometric tension recording has been described in full elsewhere [10,21]. Two parallel stainless steel pins were introduced through the lumen of the vascular segment: one was fixed to the bath wall, and the other connected to a force transducer (Grass FTO3C; Quincy, MA, USA); this, in turn, was connected to a model 7D Grass polygraph. For EFS experiments, segments were mounted between two platinum electrodes 0.5 cm apart and connected to a stimulator (Grass, model S44) modified to supply adequate current strength. Segments were suspended in an organ bath containing 5 mL of KHS at 37°C and continuously bubbled with a 95% O_2_ to 5% CO_2_ mixture (pH of 7.4). Some experiments were performed in endothelium-denuded segments to eliminate the main source of vasoactive substances, including endothelial NO. This avoided possible actions by different drugs on endothelial cells that could lead to misinterpretation of results. Endothelium was removed by gently rubbing the luminal surface of the segments with a thin wooden stick. The segments were subjected to a tension of 0.5 g, which was readjusted every 15 min during a 90-min equilibration period before drug administration. After this, vessels were exposed to 75 mmol/L KCl to check their functional integrity. After a washout period, the presence/absence of vascular endothelium was tested by the ability/inability of 10 μmol/L acetylcholine (ACh) to relax segments precontracted with NA (1 μmol/L).

Vasodilator response to ACh (0.1 nmol/L- 10 μmol/L) was tested in endothelium-intact arteries precontracted with NA (1μmol/L) from all experimental groups.

Frequency-response curves to EFS (1, 2, 4 and 8 Hz) were performed in endothelium-intact and endothelium-denuded mesenteric segments from all experimental groups. The parameters used for EFS were 200 mA, 0.3 ms, 1–8 Hz, for 30 s with an interval of 1 min between each stimulus [10], the time required to recover basal tone. Two successive frequency-response curves separated by 1-hour intervals produced similar contractile responses. To evaluate the neural origin of the EFS-induced contractile response, the nerve impulse propagation blocker, tetrodotoxin (TTX, 0.1 μmol/L) was added to the bath 30 min before the second frequency-response curve was performed.

To determine the participation of the adrenergic component of sympathetic innervation in EFS-induced responses in endothelium-denuded segments from control and pregnant rats, 1 μmol/L phentolamine, an alpha-adrenoceptor antagonist was added to the bath 30 min before performing the frequency-response curve. Additionally, the vasoconstrictor response to exogenous NA (1 nmol/L- 10 μmol/L) was tested in segments from both experimental groups.

The method to deplete sympathetic innervation has been used previously by our group in this artery [22]. Briefly, endothelium-denuded mesenteric segments from control and pregnant rats were incubated at room temperature for 10 minutes in KHS (NaHCO3 and NaH2PO4 were omitted, unbuffered solution) containing 0.02 mmol/L glutathione and 1.46 mmol/L of the neurotoxin 6-hydroxydopamine (6-OHDA). The pH of this solution was adjusted to 4.9 with 0.05 mmol/L NaOH and then the solution was covered with paraffin oil. Subsequently, the arteries were immersed in normal KHS and EFS-induced contraction experiments were performed. After EFS-contraction experiments mesenteric segments from control and pregnant rats were exposed to 75 mmol/L KCl to check that their functional integrity was not affected by 6-OHDA.

To study the possible participation of sensory innervation in EFS-induced responses in endothelium-denuded segments from control and pregnant rats, 0.5 μmol/L CGRP (8–37), a CGRP receptor antagonist was added to the bath 30 min before performing the second frequency-response curve.

To analyse the participation of NO in the EFS-induced response in endothelium-denuded segments from control and pregnant rats, 0.1 mmol/L N^ω^-nitro-L-arginine methyl ester (L-NAME), a non-specific inhibitor of nitric oxide synthase (NOS), was added to the bath 30 min before performing the second frequency-response curve. The vasodilator response to the NO donor, diethylamine NONOate (DEA-NO, 0.1 nmol/L-0.1 mmol/L) was determined in NA-precontracted arteries from both experimental groups. To assess the participation of superoxide anion in DEA-NO-induced vasodilation, 0.1 mmol/L tempol, a superoxide anion scavenger, was added to the bath before the frequency-response curve to DEA-NO was performed.

### Noradrenaline release

Endothelium-denuded mesenteric segments from control and pregnant rats were preincubated for 30 min in 5 mL of KHS at 37°C and continuously gassed with a 95% O_2_-5% CO_2_ mixture (stabilisation period). This was followed by two washout periods of 10 min in a bath of 0.4 mL of KHS. Then, the medium was collected to measure basal release. Next, the organ bath was refilled, and cumulative EFS periods of 30 s at 1, 2, 4 and 8 Hz were applied at 1 min intervals. Afterwards, the medium was collected to measure EFS-induced neurotransmitter release. The EFS-induced NA release was calculated by subtracting basal NA release from that evoked by EFS. Mesenteric segments were weighed in order to normalise the results.

NA release was measured using the Noradrenaline Research EIA (Labor Diagnostica Nord, Gmbh and Co., KG, Nordhon, Germany). The assay was performed following the manufacturer’s instructions. Results were expressed as ng NA/mL mg tissue.

### Nitric Oxide release

NO release was measured using fluorescence emitted by the fluorescent probe 4,5-diaminofluorescein (DAF-2) [23]. Endothelium-denuded mesenteric arteries from control and pregnant rats were subjected to a 60-minute equilibration period in HEPES buffer (in mmol/L: NaCl 119; HEPES 20; CaCl_2_ 1.2; KCl 4.6; MgSO_4_ 1; KH_2_PO_4_ 0.4; NaHCO_3_ 5; glucose 5.5; Na_2_HPO_4_ 0.15; pH 7.4) at 37°C. Arteries were incubated with 2 μmol/L DAF-2 for 30 min. The medium was then collected to measure basal NO release. Once the organ bath was refilled, cumulative EFS periods of 30 s at 1, 2, 4 and 8 Hz were applied at 1 min intervals. Afterwards, the medium was collected to measure EFS-induced NO release. The fluorescence of the medium was measured at room temperature using a spectrofluorometer (LS50 Perkin Elmer Instruments, FL WINLAB Software, Whaltmann, MA, USA) with excitation wavelength set at 492 nm and emission wavelength at 515 nm. Mesenteric segments were weighed in order to normalize the results.

The EFS-induced NO release was calculated by subtracting basal NO release from that evoked by EFS. Also, blank samples were collected in the same way from segment-free medium in order to subtract the background emission. Some assays were performed in the presence of 0.1 mmol/L L-NAME, 0.1 mmol 7-nitroindazol (7-NI), the specific nNOS inhibitor, 1 μmol/L 1400W, the specific iNOS inhibitor, or 0.1 μmol/L TTX. The amount of NO released was expressed as arbitrary units/mg tissue.

### Detection of O_2_^.-^

O_2_^.-^ levels were measured using lucigenin chemiluminescence [24]. Endothelium-denuded mesenteric segments from control and pregnant rats were rinsed in KHS for 30 min, equilibrated for 30 min in HEPES buffer at 37°C, transferred to test tubes that contained 1 mL HEPES buffer (pH 7.4) with lucigenin (5 μmol/L) and then kept at 37°C. The luminometer was set to report arbitrary units of emitted light; repeated measurements were collected for 5 min at 10 s intervals and averaged. 4,5-Dihydroxy-1,3-benzene-disulphonic acid “Tiron” (10 mmol/L), a cell-permeant, non-enzymatic O_2_^.-^scavenger, was added to quench the O_2_^.-^dependent chemiluminescence. Some segments were preincubated with 0.3 mmol/L apocinin, a NADPH oxidase inhibitor, or 0.1 mmol/L allopurinol, a xanthine oxidase inhibitor. Also, blank samples were collected in the same way without mesenteric segments to subtract background emission.

### nNOS and P-nNOS Expression

Western blot analysis of nNOS and phosphorylated nNOS (P-nNOS) expression was performed in endothelium-denuded mesenteric segments from control and pregnant rats, as previously described [23,24]. For these experiments, we used mouse monoclonal nNOS antibody (1:1000, Abcam, Cambridge, UK), rabbit polyclonal phospho1417-nNOS antibody (1:1000, Abcam, Cambridge, UK), and monoclonal anti-β-actin-peroxidase antibody (1:50000, Sigma-Aldrich, Spain). Rat brain homogenates were used as a positive control.

### Drugs used

L-NA hydrochloride, ACh chloride, diethylamine NONOate diethylammonium salt, CGRP (8–37), TTX, L-NAME hydrochloride, 7-NI, 1400W, phentolamine, apocinin, allopurinol, lucigenin, tiron, tempol and DAF-2 (Sigma-Aldrich, Madrid, Spain) were used. Stock solutions (10 mmol/L) of drugs were made in distilled water, except for NA, which was dissolved in a NaCl (0.9%)-ascorbic acid (0.01% w/v) solution. These solutions were kept at -20°C and appropriate dilutions were made in KHS on the day of the experiment.

### Data analysis

The responses elicited by EFS and NA were expressed as a percentage of the initial contraction elicited by 75 mmol/L KCl for comparison between control and pregnant rats. The relaxation induced by ACh or DEA-NO was expressed as a percentage of the initial contraction elicited by NA (Control: 1373 ± 178.4 mg; pregnant: 1366 ± 126.5 mg; P>0.05). For concentration-response curves, non-linear regression and Emax and—log EC50 were performed. Results are given as mean ± S.E.M. Statistical analysis was done by comparing the curve obtained in the presence of the different substances with the previous or control curve by means of repeated measure analysis of variance (ANOVA) followed by the Bonferroni *post-hoc* test using GraphPad Prism 5.0 software (CA, USA). Some results were expressed as differences of area under the curve (dAUC) for EFS obtained in segments from control and pregnant rats. AUC were calculated from the individual concentration-response plots. For dAUC, NO, O_2_^.-^ and NA release data, the statistical analysis was done using one-way ANOVA followed by Newman-Keuls’ *post-hoc* test. P<0.05 was considered significant.

## Results

### Vasomotor response to KCl

In endothelium-intact mesenteric segments, the vasoconstrictor response to 75 mmol/L KCl was similar in mesenteric segments from control and pregnant rats (Control: 1421 ± 114 mg; pregnant; 1538 ± 67.3 mg; P>0.05). Endothelium removal did not alter KCl-induced vasoconstriction (Control: 1677 ± 63.5 mg; pregnant: 1788 ± 54 mg; P>0.05).

### Vasodilator response to ACh

**Fig 1:**
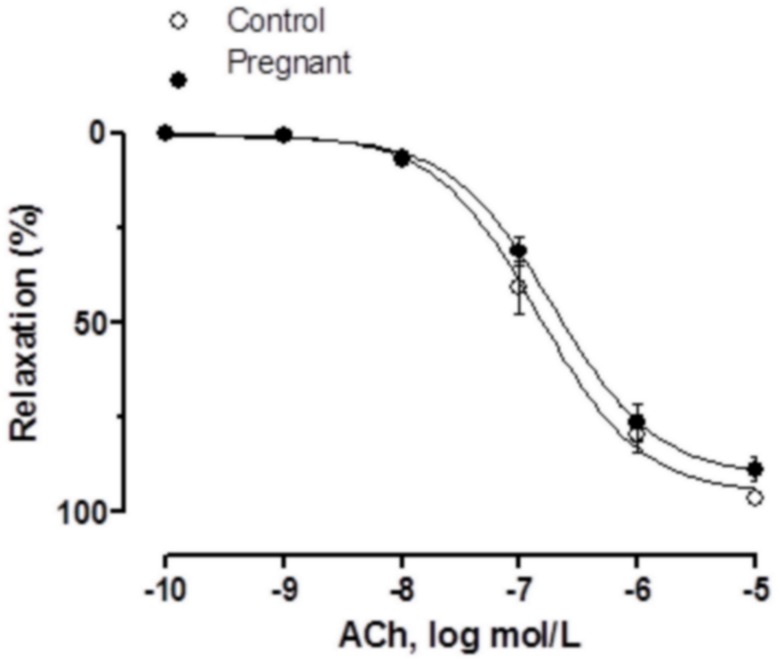
Vasodilator response to ACh. ACh-induced vasodilation in endothelium-intact mesenteric segments from control and pregnant rats. Results (mean±SEM) were expressed as a percentage of the previous tone elicited by exogenous NA. n = 6 animals each group.

Vasodilator response to ACh was similar in NA-precontracted segments from both experimental groups (Fig 1, Table 1).

**Table 1:** E_max_ (%) and log EC_50_ (mmol/L) values of vasodilator responses to Ach and DEA-NO or vasoconstrictor responses to NA in mesenteric arteries from control and pregnant rats.

	Control		Pregnant	
	E_max_	-logEC_50_	E_max_	-logEC_50_
ACh	95.09±3.07	6.84±0.08	90.58±2.76	6.72±0.07
NA	167.4±10.2	7.26±0.12	129.7±8.44*	7.23±0.2
DEA-NO	101.8±2.11	6.83±0.06	94.27±3.15	7.21±0.11

Results are expressed as mean±SEM.

*P<0.05 vs. Control.

n = 7 animals each group.

### Vascular responses to EFS

**Fig 2:**
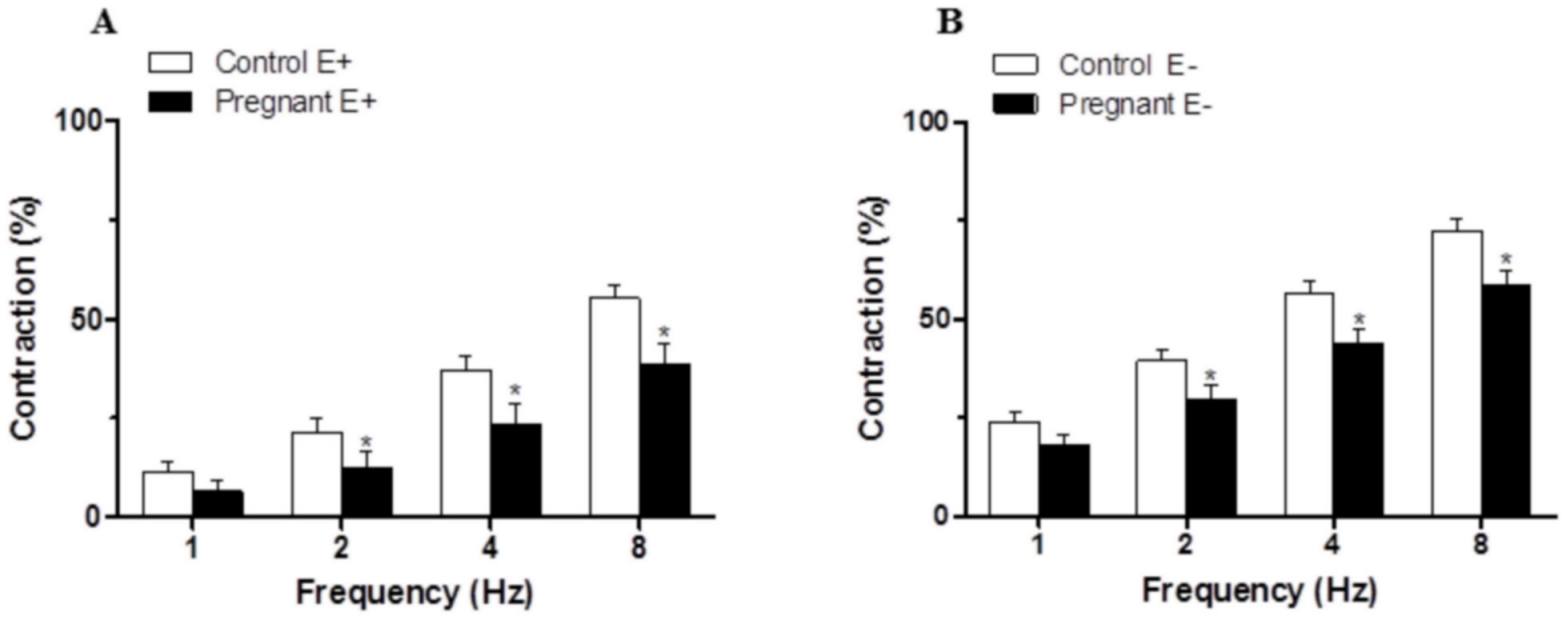
Vasoconstrictor response to EFS. EFS-induced vasoconstriction in endothelium intact (A) and endothelium-denuded (B) mesenteric segments from control and pregnant rats. Results (mean±SEM) were expressed as a percentage of the initial contraction elicited by KCl. ANOVA P<0.05 Control vs. pregnant in both endothelium intact (A) and endothelium-denuded (B). *P<0.05 vs. control animals at each frequency (Bonferroni test). n = 10 animals each group.

The application of EFS induced a frequency-dependent contractile response in both endothelium-intact and endothelium-denuded mesenteric segments from both experimental groups. This vasoconstriction was lower in segments from pregnant rats compared to control animals (Fig 2A and 2B). Endothelium removal increased the EFS-induced contractile response to a similar extent in segments from control and pregnant rats (Fig 2A and 2B, Table 2). EFS-induced contractions were practically abolished by preincubation with neurotoxin TTX (0.1 μmol/L), indicating the neuronal origin of the factors inducing this response (Table 3).

**Table 2:** EFS potentiation after endothelium removal in superior mesenteric artery from control and pregnant rats.

	1 Hz	2 Hz	4 Hz	8 Hz
Control	12.51±2.61	18.19±2.79	19.38±3.05	17.23±3.32
Pregnant	11.55±2.92	17.39±3.85	20.26±4.37	19.79±4.26

Percentage (%) of potentiation in EFS-induced contraction after endothelium removal. Calculations are performed taking KCl-induced contraction as 100% of the contractile response. Results are expressed as mean ± SEM. n = 10 animals each group.

**Table 3:** Effect of preincubation with tetrodotoxin (TTX, 0.1μmol/L) or 6-hydroxydopamine (6-OHDA, 1.46 mmol/L) on the frequency—contraction curves performed in mesenteric segments of control and pregnant rats.

	1 Hz	2 Hz	4 Hz	8 Hz
**Control**	21.31±2.23	34.10±2.56	52.41±2.22	69.28±3.91
TTX	0	0	0	0.5±0.06
6-OHDA	0	0	0.1±0.02	0.2±0.04
**Pregnant**	16.42±2.72	25.9±3.58	39.28±4.04	53.75±3.99
TTX	0	0	0	0.4±0.05
6-OHDA	0	0	0.1±0.01	0.3±0.05

Results (means ± S.E.M.) are expressed as percentages of the response elicited by 75 mM KCl; zeros are used when contraction was not detected. n = 7 animals each group.

### Effect of pregnancy on the sympathetic component of vascular responses to EFS in endothelium-denuded mesenteric segments

**Fig 3:**
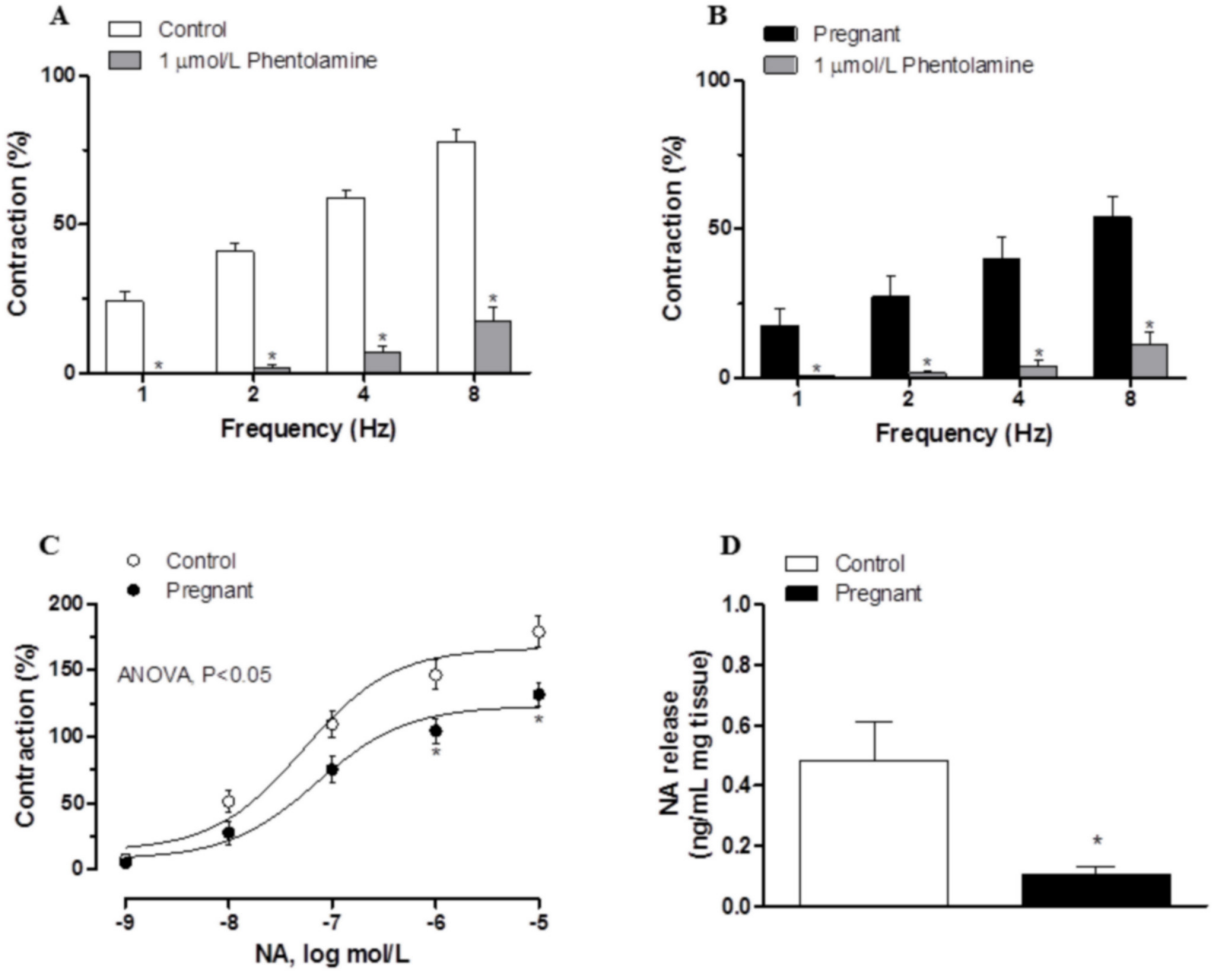
Effect of pregnancy on sympathetic innervation function. Effect of preincubation with 1 μmol/L phentolamine on vasonstriction response induced by EFS in endothelium-denuded mesenteric segments from control (A) and pregnant rats (B). Results (mean±SEM) were expressed as a percentage of the initial contraction elicited by KCl. ANOVA P<0.05 vs. conditions without phentolamine in both experimental groups. *P<0.05 vs. conditions without phentolamine at each frequency (Bonferroni test). n = 8 animals per group. (C) Vasoconstriction response to NA in segments of control and pregnant rats. Results (mean±SEM) were expressed as a percentage of the initial contraction elicited by KCl. ANOVA P<0.05 Control vs. pregnant. *P<0.05 vs. control animals at each concentration (Bonferroni test). n = 8 animals per group. (D) EFS-induced NA release in mesenteric segments of control and pregnant rats. Results expressed as ng NA/mL mg tissue. *P<0.05 vs. Control. n = 6 animals per group.

Preincubation with the alpha-adrenergic antagonist phentolamine (1 μmol/L) decreased the vasoconstrictor response induced by EFS in segments from both experimental groups (Fig 3A and 3B). This decrease was lower in arteries from pregnant rats (Table 4). The phentolamine-resistant contractile response to EFS was lower in segments from pregnant rats (Table 5). Preincubation with 6-OHDA practically abolished the EFS-induced contraction in segments from control and pregnant rats (Table 3). After 6-OHDA preincubation, vasomotor response to KCl was similar in mesenteric segments from both experimental groups (Control: 1501 ± 95.4 mg; pregnant; 1622 ± 109 mg; P>0.05).

**Table 4:** Effect of preincubation with phentolamine (0.1 μmol/L) on the frequency—contraction curves performed in mesenteric segments from control and pregnant rats.

	1 Hz	2 Hz	4 Hz	8 Hz
**Control**	24.1±3.23	41.06±2.84	59.14±2.22	77.76±3.90
Phentolamine inhibition	23.79±4.01*	38.88±3.08*	52.90±2.74*	62.63±3.47*
**Pregnant**	17.53±3.71	27.17±5.84	40.14±5.42	54.03±5.96
Phentolamine inhibition	16.96±5.55*	25.54±6.42* #	36.13±6.39* #	42.97±4.12* #

Percentage (%) of inhibition in EFS-induced contraction after preincubation with phentolamine. Calculations are performed taking KCl-induced contraction as 100% of the contractile response. Results are expressed as mean ± SEM.

*P<0.05 vs. conditions without phentolamine at each frequency (Bonferroni test).

^#^P<0.05 vs. Control.

n = 8 animals per group.

**Table 5:** Remnant vasoconstriction after preincubation with phentolamine (0.1 μmol/L) on the frequency—contraction curves performed in mesenteric segments of control and pregnant rats.

	1 Hz	2 Hz	4 Hz	8 Hz
**Control**	24.1±3.23	41.06±2.84	59.14±2.22	77.76±3.90
Phentolamine Remnant	0.40±0.19*	2.17±0.88*	7.98±1.38*	18.35±5.54*
**Pregnant**	17.53±3.71	27.17±5.84	40.14±5.42	54.03±5.96
Phentolamine Remnant	0.61±0.4*	1.63±0.56*	3.72±2.06* #	9.97±3.63* #

Results (means ± S.E.M.) are expressed as percentages of the response elicited by 75 mM KCl.

*P<0.05 vs. conditions without phentolamine at each frequency (Bonferroni test).

^#^P<0.05 vs. Control.

n = 8 animals per group.

The contractile response elicited by exogenous NA (1 nmol/L-10 μmol/L) was reduced in segments from pregnant rats (Fig 3C, Table 1). Additionally, EFS-induced NA release was lower in segments from pregnant rats (Fig 3D).

### Effect of pregnancy on the sensory component of vascular responses to EFS in endothelium-denuded mesenteric segments

**Fig 4:**
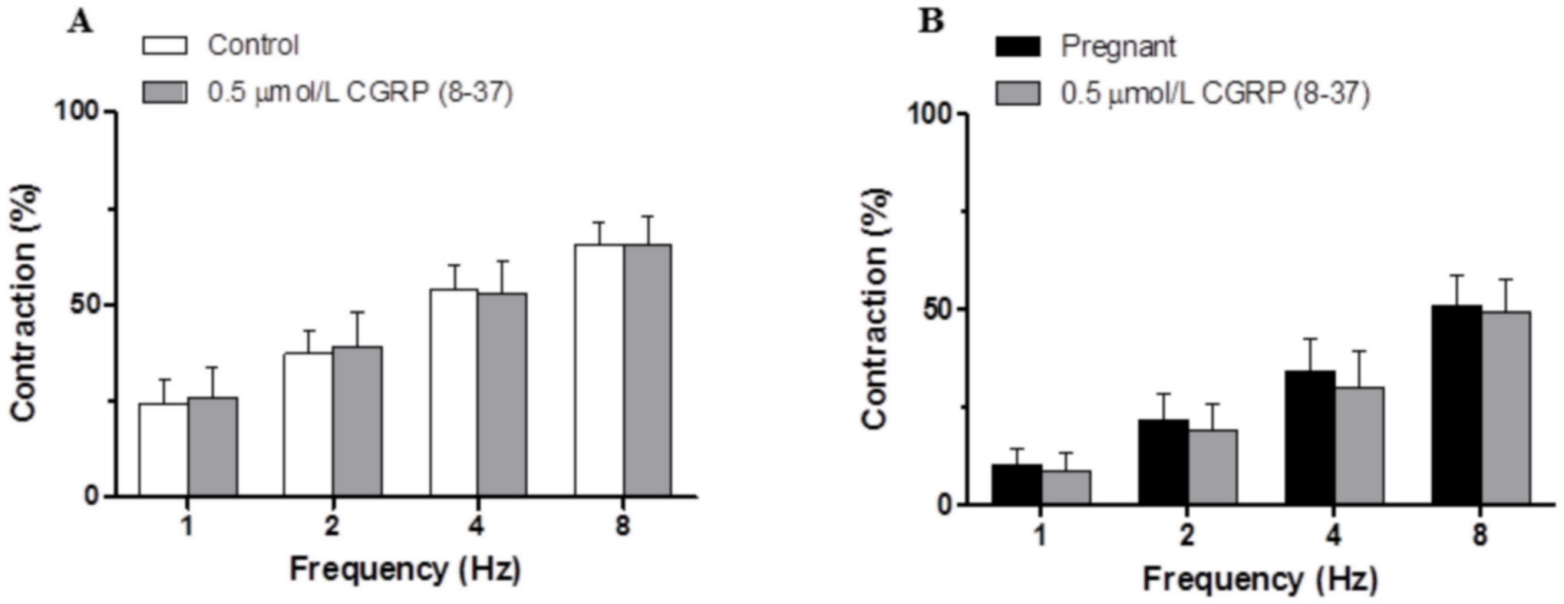
Effect of pregnancy on sensory innervation. Effect of preincubation with 0.5 μmol/L CGRP (8–37) on the vasoconstrictor response induced by EFS in mesenteric segments from (A) control and (B) pregnant rats. Results (mean±SEM) are expressed as a percentage of the previous contraction elicited by KCl. n = 8 animals per group.

Preincubation with the CGRP receptor antagonist CGRP (8–37) (0.5 μmol/L) did not alter the EFS-induced contraction in any experimental group, indicating that sensory innervation did not contribute to the observed effects (Fig 4A and 4B).

### Effect of pregnancy on the nitrergic component of vascular responses to EFS in endothelium-denuded mesenteric segments

**Fig 5:**
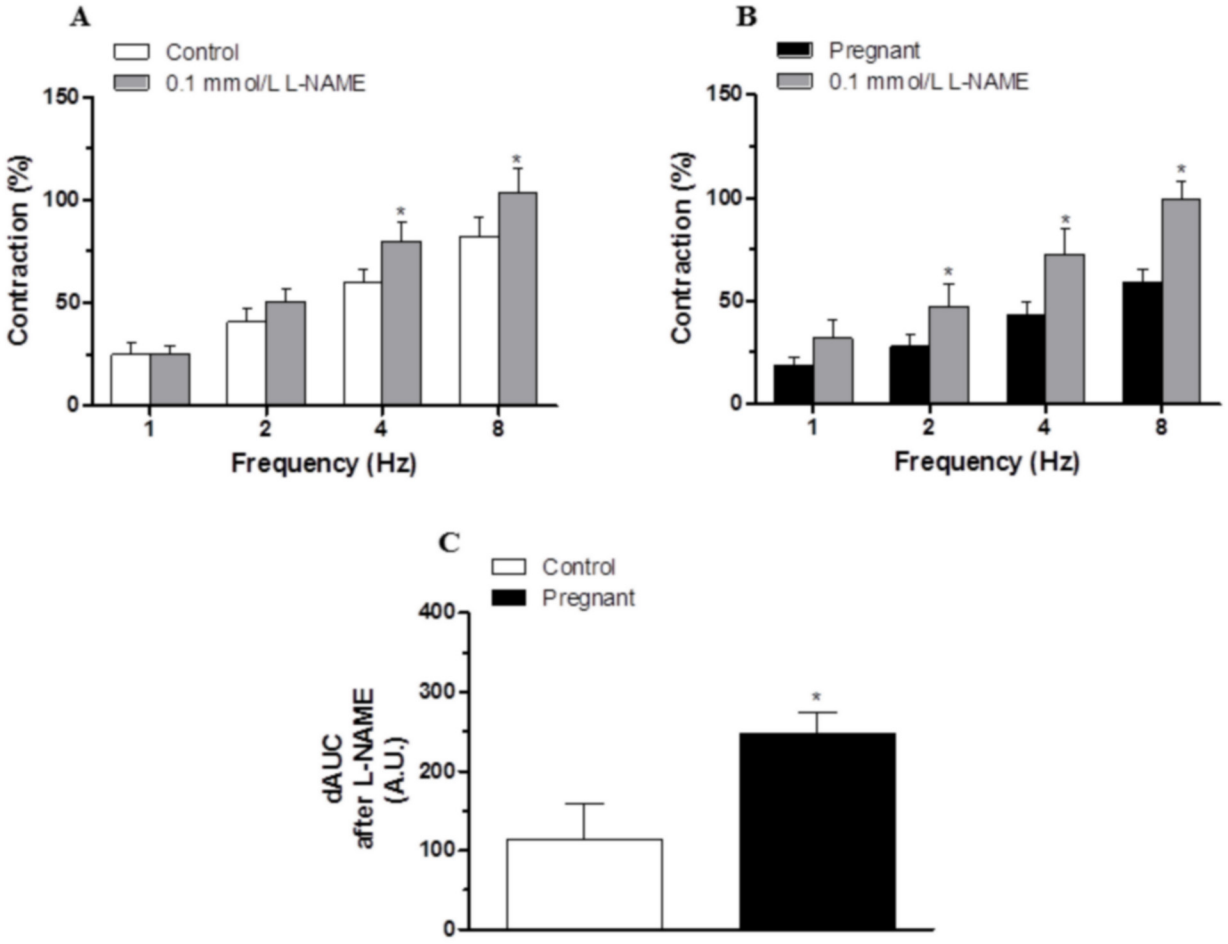
Effect of pregnancy on nitrergic innervation. Effect of preincubation with 0.1 mmol/L L-NAME on the vasoconstrictor response induced by EFS in mesenteric segments from (A) control and (B) pregnant rats. Results (mean±SEM) are expressed as a percentage of the previous contraction elicited by KCl. ANOVA P<0.05 vs. conditions without L-NAME in both experimental groups. *P<0.05 vs. conditions without L-NAME for each frequency (Bonferroni test). n = 8 animals per group. (C) Differences of area under curve (dAUC) in the absence or presence of 0.1 mmol/L L-NAME, dAUC values are expressed as arbitrary units.*P<0.05 Control vs. pregnant rats.

Preincubation with the unspecific NOS inhibitor L-NAME (0.1 mmol/L) significantly increased the EFS-contractile response at all frequencies in segments from control and pregnant rats (Fig 5A and 5B). This increase was greater in arteries from pregnant animals (Fig 5C).

**Fig 6:**
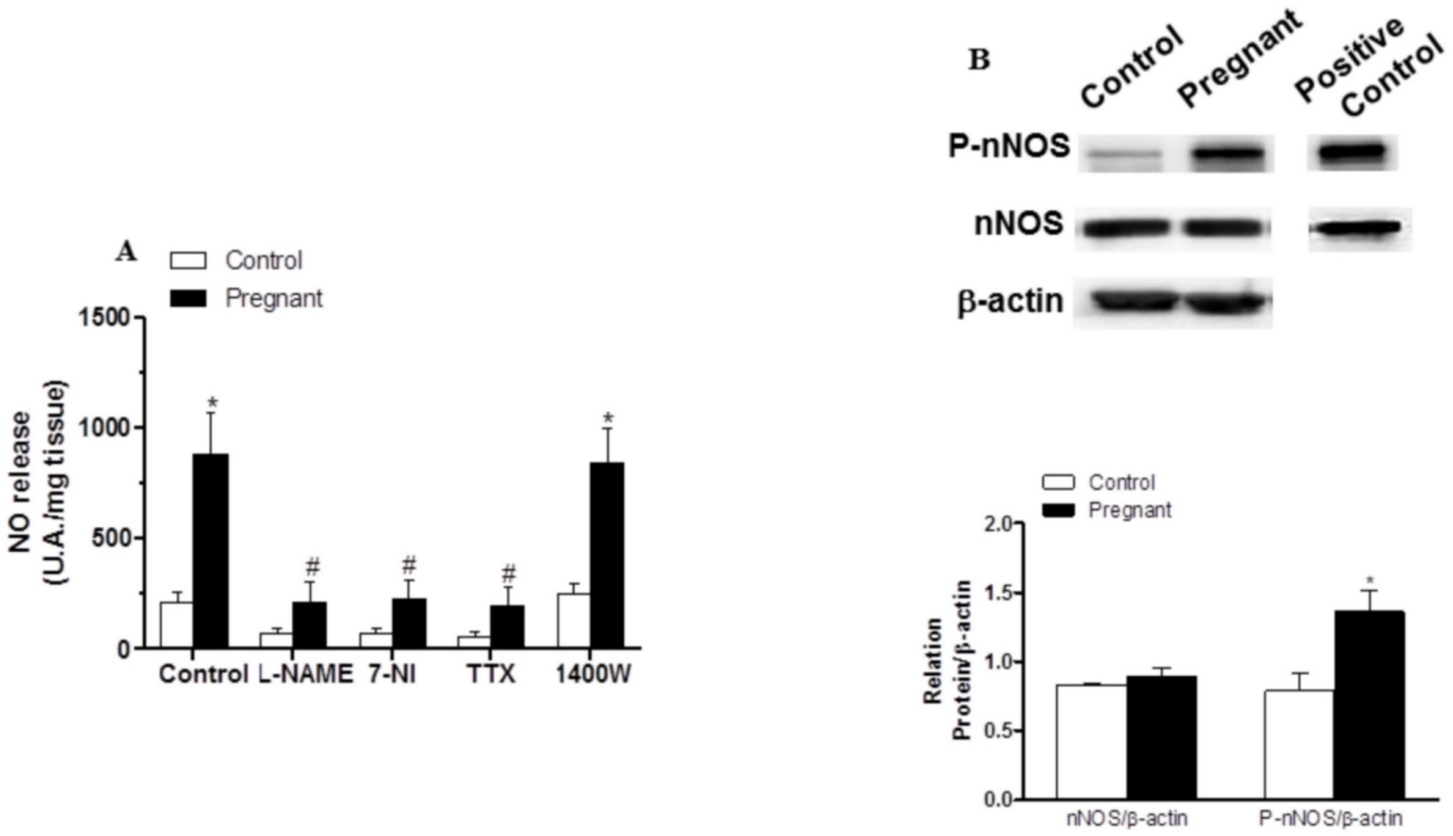
Effect of pregnancy on neuronal NO synthesis. (A) Effect of 0.1 mmo/L L-NAME or 0.1 mmol/L 7-NI or 0.1 μmol/L TTX on EFS-induced NO release in segments from control and pregnant rats. Results (mean±SEM) were expressed as arbitrary (A.U.)/mg tissue. *P<0.05 vs. Control; #P<0.05 compared with conditions without specific inhibitor; n = 8 animals per group. (B) Effect of pregnancy on nNOS and P-nNOS expression. The blot is representative of four separate segments from each group. Rat brain homogenates were used as a positive control. Lower panel shows relation between P-nNOS or nNOS expression and β-actin. Results (mean±SEM) are expressed as ratio of the signal obtained for each protein and the signal obtained for β-actin. *P<0.05 Control vs. pregnant rats.

EFS-induced NO release was significantly greater in segments from pregnant rats (Fig 6A). Preincubation with 0.1 mmol L-NAME, 0.1 mmol 7-NI or 0.1 μmol/L TTX abolished EFS-induced NO release in both experimental groups (Fig 6A), while preincubation with 1 μmol/L 1400W did not modify this release (Fig 6A). Pregnancy did not modify nNOS expression but did increase P-nNOS expression (Fig 6B).

**Fig 7:**
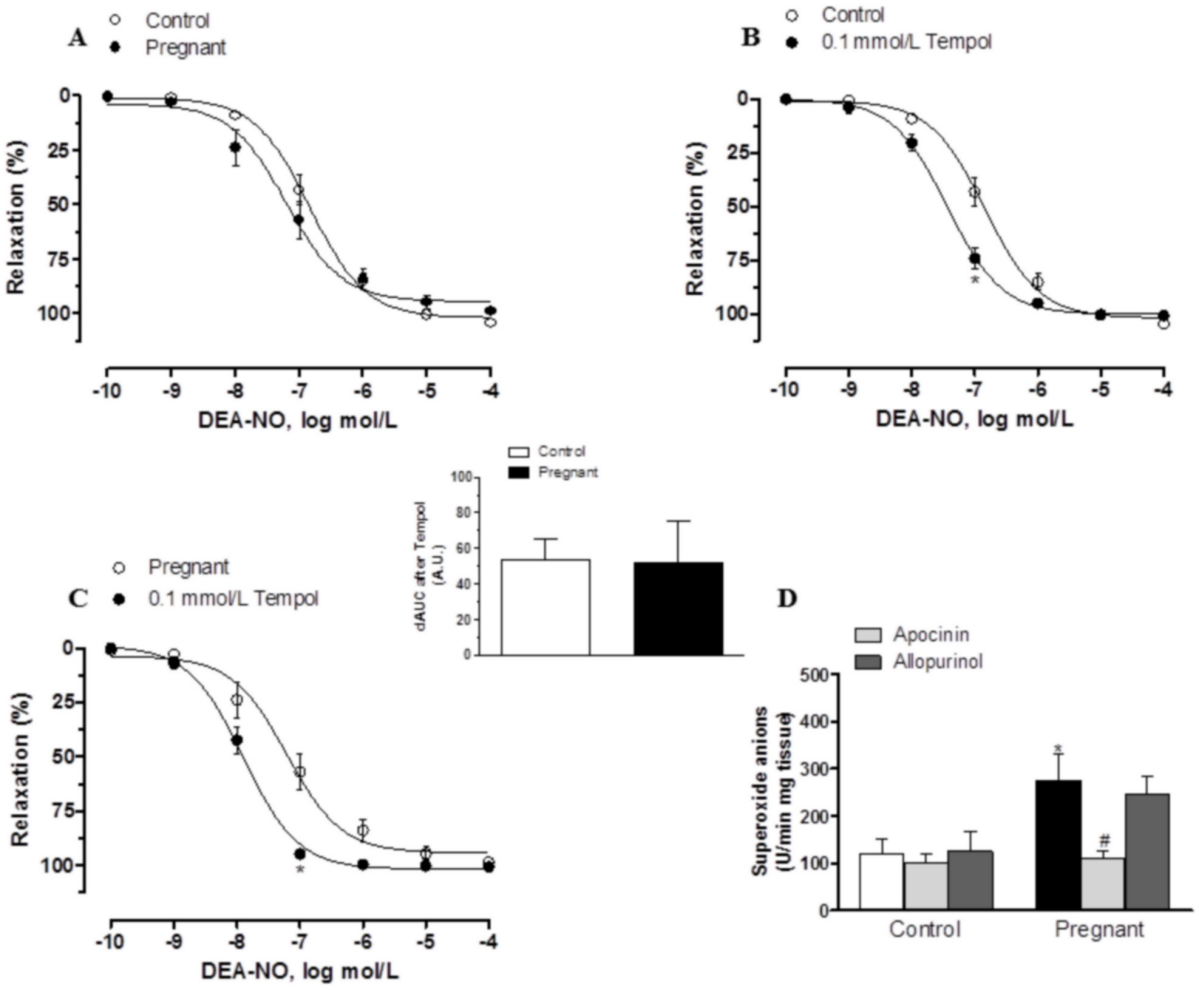
Effect of pregnancy on DEA-NO vasodilation and superoxide anion production. (A) Vasodilator response to NO donor DEA-NO in segments from control and pregnant rats. Effect of preincubation with tempol (0.1 mmol/L) on vasodilator response to NO donor DEA-NO in segments from (B) control and (C) pregnant rats. Results (mean±SEM) are expressed as a percentage of the previous tone elicited by exogenous NA. ANOVA P<0.05 vs. conditions without tempol in both experimental groups. *P<0.05 vs. conditions without tempol for each concentration (Bonferroni test). n = 7 animals per group. Insert graph shows differences of area under curve (dAUC) in the absence or presence of 0.1 mmol/L tempol; dAUC values are expressed as arbitrary units. (D) Effect of 0.3 mmol/L apocinin or 0.1 mmol/L allopurinol on superoxide anion release in mesenteric segments from control and pregnant rats. Results (mean±SEM) are expressed as chemoluminiscence units (U)/min mg tissue. *P<0.05 vs. Control; #P<0.05 compared with conditions without specific inhibitor; n = 7 animals per group.

In segments precontracted with NA, vasodilator response elicited by the exogenous NO donor DEA-NO (0.1 nmol/L- 10 μmol/L) was similar in mesenteric segments from both experimental groups (Fig 7A, Table 2). Preincubation with 0.1 mmol/L tempol augmented DEA-NO-induced vasodilation similarly in segments from control and pregnant rats (Fig 7B and 7C). On the other hand, after subtracting the lucigenin chemiluminescence obtained in the presence of tiron from that obtained in its absence, superoxide anion formation was greater in arteries from pregnant rats (Fig 7D). Preincubation with apocinin decreased superoxide anion formation significantly in mesenteric segments of pregnant rats, while allopurinol did not modify this formation in any experimental group (Fig 7D)

## Discussion

The present study shows that pregnancy diminished the vasoconstrictor response due to stimulation of perivascular innervation in mesenteric artery through an endothelium-independent mechanism. This effect is associated with a reduction in sympathetic innervation function mediated by a decreased NA release and vasoconstrictor response and an increased nitrergic function associated with increased neuronal NO release. Sensory innervation did not participate in any experimental conditions.

When analyzing the vasoconstrictor response induced by EFS in endothelium-intact segments, we observed that EFS produced a frequency-dependent contraction in segments from both control and pregnant rats that was lower in segments from pregnant rats, as previously reported [4,25,26]. The fact that in our experimental conditions the vasoconstrictor response to KCl remained unmodified by pregnancy, as has been previously reported in this artery [4,27], indicates the maintenance of vasoconstrictor capacity, ruling out possible changes in the intrinsic contractile machinery of smooth muscle cells.

It has been widely described that endothelium affects the response to several vasomotor substances, including neurotransmitters [28], and since pregnancy could affect the production of endothelial factors [29] it would consequently affect these responses. In our experimental conditions ACh-induced vasodilation was not modified by pregnancy as also reported by Ralevic and Burnstock at late-term pregnancy [4]. Endothelium removal increased vasoconstrictor response to EFS similarly in both groups, indicating that the modulating role of endothelium in the EFS vasoconstrictor response is unaltered by pregnancy so the decreased contractile response would be due to modifications in perivascular innervation function produced by an endothelium-independent mechanism. Taking into account these results, we performed the following experiments only in endothelium-denuded mesenteric segments. The abolishment of EFS-induced vasoconstriction in the presence of TTX indicates that the response to EFS is due to neurotransmitter release. Sympathetic and nitrergic innervation involvement in the vasomotor response to EFS in rat superior mesenteric artery has been widely reported [9,10], while sensory innervation participation only appears in several pathological situations [13,30,31,32,33].

A decreased participation of sympathetic innervation in splanchnic blood flow as an adaptation to pregnancy has been demonstrated previously and associated with decreased vasoconstrictor response to NA [7]. However the possibility that the NA release was also affected by pregnancy has been also suggested but not measured [3,4]. Preincubation with the alpha-adrenoceptor antagonist phentolamine significantly diminished the vasoconstrictor response to EFS in mesenteric segments from both experimental groups. This decrease was less marked in mesenteric segments from pregnant rats, indicating a decreased participation by the adrenergic component from sympathetic innervation in the lower vasoconstrictor response to EFS in pregnant rats, similarly to results described by Ralevic and Burnstock [4]. The differences in the vasoconstrictor response to EFS could be associated with changes in neurotransmitter release and/or vasomotor response to NA. The results obtained demonstrate that late pregnancy decreases the vasoconstrictor response to NA and simultaneously decreases NA release in mesenteric artery. It is well known that there is a decrease in vasomotor responses to contractile substances in pregnancy, although the mechanisms implicated are not well known. No changes in alpha-adrenoceptor affinity in pregnancy [34], decreased alpha(1)-adrenoceptor expression [35] and modifications in the regulation of Ca(2+) mobilization and Ca(2+) sensitivity in alpha(1)-adrenoceptor-mediated contractions [36] have all been described. While the decrease in NA release could be associated with changes in sympathetic nerve density due to estrogens, previous studies have report that pregnancy produces no change [37], increased [38] and decreased [39] sympathetic nerve density, depending on the tissue studied. We have reported that the remnant vasoconstriction after preincubation with phentolamine is due to the release of sympathetic cotransmitter ATP in mesenteric segments from female rats [14]. Preincubation with the neurotoxine 6-OHDA practically abolished EFS-induced contraction in both experimental groups while the remnant vasoconstriction after preincubation with phentolamine was decreased in mesenteric segments of pregnant rats, suggesting a minor role for ATP in late pregnancy. The diminished sympathetic innervation function could, by itself, explain the decreased EFS-induced vasoconstriction observed in mesenteric segments from pregnant rats. However, the participation of other neural components cannot be ruled out.

We have previously reported that sensory innervation does not participate in vasomotor response induced by EFS in control Sprague Dawley rats [14]. However, the participation of this innervation is increased in several pathological conditions such as hypertension, cirrhosis and diabetes [20,30,31,32]. Important variations in serum levels of the vasodilator CGRP have been reported in humans and rats during pregnancy [40,41]. Additionally, in rat mesenteric arteries, both early and late pregnancy are associated with a rise in the vascular sensitivity to CGRP in selected areas of the vascular bed without a concomitant increase in vascular CGRP production and release [15,16,42]. This observation led us to study the possible role of sensory innervation in the decreased EFS vasoconstriction observed in pregnant rats. Preincubation with the CGRP receptor antagonist CGRP (8–37) did not alter EFS-induced vasoconstriction in any experimental group, indicating that in late pregnancy sensory innervation does not participate in the vasoconstrictor response to EFS. This result contrasts with those of Ralevic and Burnstock [4], who observed a participation of sensory innervation in the EFS-mediated relaxation, and this response was not modified by pregnancy. Nevertheless, Lanlua *et al*. [43] reported no effect from CGRP (8–37) in the EFS-mediated relaxation of small mesenteric arteries in pregnancy.

It has also been suggested an involvement by NO in the decreased vasoconstrictor response to EFS in conductance and resistance mesenteric arteries during pregnancy [4,25] since there is increased NO production and release by endothelial cells. However, the fact that the unspecific inhibition of NO synthesis also affects neuronal NO does not rule out the participation of nitrergic perivascular innervation, especially when NO released from nitrergic innervation has been demonstrated to have a vasodilator role that contributes to decreasing the vasomotor response to EFS [10]. Previously, we have demonstrated that both the non-selective NOS inhibitor L-NAME and the specific nNOS inhibitor 7-NI decrease EFS-induced NO release to a similar extent [23]. However, in vascular reactivity experiments, preincubation with 7-NI also decreased the vasoconstrictor response to NA, making the analysis of EFS-induced contractions very complex, and leading to result misinterpretation [23,44]. For that reason, we used L-NAME instead of 7-NI in vascular reactivity experiments. Vasoconstriction induced by EFS was increased in L-NAME incubated segments from both groups of rats, suggesting an involvement by NO in this response, as previously described in this rat strain [10]. The greater effect of L-NAME observed in segments from pregnant rats suggests an increased role of neuronal NO in EFS-induced vasoconstriction related to increases in NO release and/or increases in the vasodilator response. EFS-induced NO release was higher in segments from pregnant animals, confirming our hypothesis. Production of NO in neural tissue can have two sources: nNOS and iNOS [22,44,45]. EFS-induced NO release was abolished by preincubation with L-NAME, 7-NI or TTX in both experimental groups, confirming that EFS induced NO release from nitrergic innervation. Additionally, in the present study, the specific iNOS inhibitor 1400W did not change EFS-induced NO release in segments from the two experimental groups, ruling out the participation of this inducible isoform in our experimental approach, as previously reported [22,46].

The differences in NO release observed in our experimental conditions could be dependent on nNOS expression and/or activity. We found that nNOS protein expression was not modified by pregnancy, whereas P-nNOS, the active form of nNOS was increased, suggesting increased nNOS activity. Estrogens are reported to regulate constitutive nitric oxide synthases and this regulation could be implicated in changes occurring in pregnancy [47]. Additionally Martini *et al*. [48] have reported an increase in nNOS expression correlated with increased female sex hormone levels, suggesting a complex action by female sex hormones in nNOS expression; therefore the increased nNOS activity could be due to the estrogens as has been reported previously [49].

Thus, increased NO release participates in the diminished response to EFS in segments from pregnant rats. However, altered smooth muscle cell sensitivity to NO cannot be ruled out since measurements of oxidative stress markers in maternal blood and urine show that pregnancy itself is a state of oxidative stress due to the high metabolic activity of the placenta and maternal metabolism [50], and this could alter NO bioavailability. First, we confirmed that superoxide anion production is increased in mesenteric segments from pregnant rats. Oxidative stress in pregnancy is regulated differently depending on the organ analysed [51]. It is well known that NADPH oxidase is the main source of superoxide anion in vessels [52]. Taking this into account, we have measured superoxide anion production in the presence of the NADPH oxidase inhibitor apocinin and the xanthine oxidase inhibitor allopurinol. Preincubation with apocinin significantly decreased superoxide anion production only in mesenteric segments of pregnant rats, but allopurinol did not modify superoxide anion production in any experimental group. These results confirm NADPH oxidase as the principal source of oxidative stress in superior mesenteric artery in late pregnancy. However, the vasodilator response to DEA-NO was similar in segments from both experimental groups. Additionally, preincubation with the superoxide anion scavenger tempol increased vasodilator response to DEA-NO to a similar extent in mesenteric segments from both control and pregnant rats. These results suggest that, despite the increased superoxide anion levels observed, the oxidation process is unable to affect the NO metabolism enough to significantly affect vasodilator response.

In conclusion, our results show that neural control of mesenteric vasomotor tone was altered by pregnancy. Diminished sympathetic and enhanced nitrergic components both contributed to a decreased vasoconstriction response to EFS. The sensory innervation did not participate in these modifications. All these changes indicate the simultaneous implication of different components of perivascular innervation in vascular adaptations to pregnancy.
